# Supplementary material for: Assembly of the Complete Mitochondrial Genome of Pereskia aculeata Revealed That Two Pairs of Repetitive Elements Mediated the Recombination of the Genome
Source: Int J Mol Sci. 2023 May 6;24(9):8366. doi: 10.3390/ijms24098366 (PMC10179450; doi:10.3390/ijms24098366)

Supplementary figure legends

Fig. S1 Sequencing depth of coverage based on Nanopore long-reads. The abscissa indicates the location of the contig, and the ordinate indicates the sequencing depth. The average sequencing depth of the mitochondrial genome was about 75 ×.

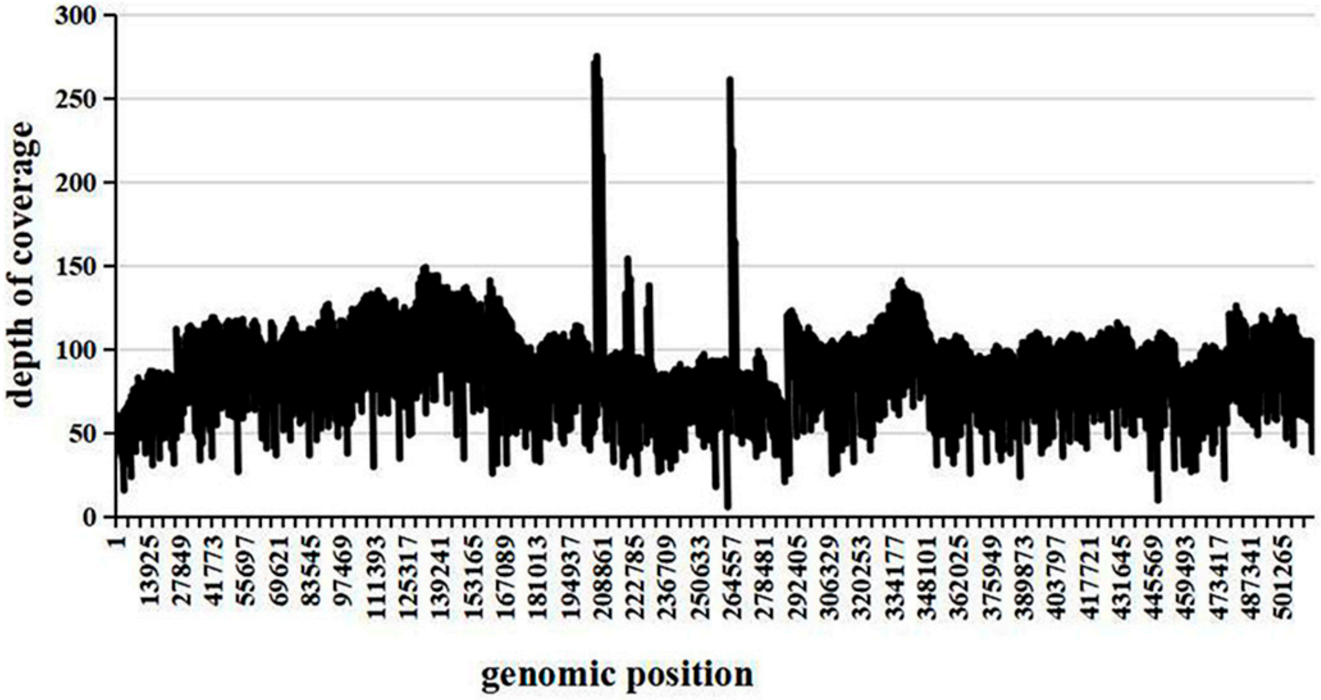

Supplement: Supplementary file 1 [file ijms-24-08366-s001.zip › FigureS1.pdf]
